# Identification of genetic loci associated with higher resistance to pancreas disease (PD) in Atlantic salmon (*Salmo salar* L.)

**DOI:** 10.1186/s12864-020-06788-4

**Published:** 2020-06-03

**Authors:** Borghild Hillestad, Shokouh Makvandi-Nejad, Aleksei Krasnov, Hooman K. Moghadam

**Affiliations:** 1 Benchmark Genetics Norway AS, Sandviksboder 3A, N-5035 Bergen, Norway; 2 Norwegian Veterinary Institute, P.O. Box 750, Sentrum, N-0106 Oslo, Norway; 3 Division of Aquaculture, Norwegian Institute of Fisheries and Aquaculture (Nofima), P.O. Box 6122, Muninbakken 9-13, Breivika, Langnes, N-9291 Tromsø, Norway

**Keywords:** Atlantic salmon, Pancreas disease, Transcriptome, GWAS, Heritability, Breeding

## Abstract

**Background:**

Pancreas disease (PD) is a contagious disease caused by *salmonid alphavirus* (SAV) with significant economic and welfare impacts on salmon farming. Previous work has shown that higher resistance against PD has underlying additive genetic components and can potentially be improved through selective breeding. To better understand the genetic basis of PD resistance in Atlantic salmon, we challenged 4506 smolts from 296 families of the SalmoBreed strain. Fish were challenged through intraperitoneal injection with the most virulent form of the virus found in Norway (i.e., SAV3). Mortalities were recorded, and more than 900 fish were further genotyped on a 55 K SNP array.

**Results:**

The estimated heritability for PD resistance was 0.41 ± 0.017. The genetic markers on two chromosomes, ssa03 and ssa07, showed significant associations with higher disease resistance. Collectively, markers on these two QTL regions explained about 60% of the additive genetic variance. We also sequenced and compared the cardiac transcriptomics of moribund fish and animals that survived the challenge with a focus on candidate genes within the chromosomal segments harbouring QTL. Approximately 200 genes, within the QTL regions, were found to be differentially expressed. Of particular interest, we identified various components of immunoglobulin-heavy-chain locus B (IGH-B) on ssa03 and immunoglobulin-light-chain on ssa07 with markedly higher levels of transcription in the resistant animals. These genes are closely linked to the most strongly QTL associated SNPs, making them likely candidates for further investigation.

**Conclusions:**

The findings presented here provide supporting evidence that breeding is an efficient tool for increasing PD resistance in Atlantic salmon populations. The estimated heritability is one of the largest reported for any disease resistance in this species, where the majority of the genetic variation is explained by two major QTL. The transcriptomic analysis has revealed the activation of essential components of the innate and the adaptive immune responses following infection with SAV3. Furthermore, the complementation of the genomic with the transcriptomic data has highlighted the possible critical role of the immunoglobulin loci in combating PD virus.

## Background

Pancreas disease (PD) is a severe contagious disease of farmed Atlantic salmon (*Salmo salar*) and rainbow trout (*Oncorhynchus mykiss*), affecting fish during the sea-water phase of the production. The aetiological agent, *salmonid alphavirus* (SAV), is a single-stranded RNA virus belonging to the *Togaviridae* family [1], and it has become a pathogen of high economic concern in the salmon farming countries such as Norway, Scotland and Ireland. So far, six different subtypes of this virus, SAV1 to SAV6, have been identified [2–4], where outbreaks caused by SAV3 have only been reported in the Norwegian sea-waters to date [5–7]. SAV3 was first detected and described, at its genomic details, based on the isolates collected from the west coast of Norway in 2003 and 2004 [8] where now constitute an endemic region for this disease. Since 2007, PD has become a notifiable disease in Norway, and strict national regulations have been in place for more efficient confinement of the spread of the virus. Since then, the number of PD cases throughout Norway, due to SAV3 infection, has remained relatively constant with about 100 outbreaks per year [9].

The economic losses due to PD outbreaks can be substantial. Based on an economic model, the estimated direct associated cost in 2007 with a PD outbreak for 500,000 smolts in Norway was about 14.4 million Norwegian Kroners (NOK; approximately €1.45 M) [10]. The same study also found that the saleable biomass due to this disease was reduced by 70% and the production costs increased with 6 NOK per kg. An updated analysis from the data, based on 2013 sale prices, suggested that the direct cost of PD outbreak for 1,000,000 smolts to be about 55.4 M NOK (approximately €5.53 M) [11]. These figures suggest that although different methods of prevention, such as vaccination, improving management and optimised production conditions, have caused the mortality during an outbreak to decline, still, the financial losses due to PD infections have increased.

SAV usually infects salmon at the smolt stage during the first year in the sea. Clinical manifestation of PD infection might include sudden loss of appetite and lethargy, reduction in growth, abnormal swimming behaviour and increased mortality [12]. Mortality due to PD infection can vary from negligible to very high, with an expected average mortality of around 7%, based on data collected from 2006 to 2008 [13]. Histopathological examination of infected animals often exhibits loss of exocrine pancreatic tissue, cardiac degeneration, inflammation and subsequent degeneration and inflammation of the skeletal muscle [14]. Following infection, mainly if the infection occurs during the later stages of production, a significant reduction in growth and deterioration in the feed conversion ratio can be expected [15]. Further, the fish that survive the outbreak can become more susceptible to other pathogens and secondary infections [16].

So far, a few studies have aimed to evaluate a host immune response to infection with PD [17]. It has been demonstrated that following an infection, the virus will stimulate the expression of innate immunity through pattern recognition receptors (PRR) such as toll-like receptors (TLRs) and RNA helicases. Subsequently, this stimulation triggers antiviral effectors and the type I interferons (IFN I) signalling pathway, where the most significant changes in the gene expression response of a host can be observed in the infected heart. The stimulation of these genes and genetic pathways, where in turn, further enhances the innate immune responses [18, 19]. Subsequent inflammation in the affected tissues, triggered by the products of immune-related genes like chemokines and cytokines is then expected [17]. Activation of adaptive immune responses and production of neutralizing antibodies takes place following PD infection [20–22].

A possible solution and a common practice to enhance resistance against PD is vaccination. Several vaccines including a multivalent vaccine by MSD Animal Health (AquaVac® PD_t_), a monovalent vaccine by PHARMAQ (ALPHA JECT micro® 1PD) and a DNA vaccine by Elanco (Clynav) are a few examples. Although the efficiency of vaccines has been questioned regarding their failure to eradicate the disease, in addition to their high costs, various side effects and negative impacts on slaughter quality, vaccination helps to reduce the mortality and alleviate some of the associated signatures of the infection, such as weight loss and feed conversion rate [23].

An early indication of a possible genetic basis for PD resistance was reported through epidemiological observations in PD response between different strains of Atlantic salmon [16, 24, 25]. Subsequent works have shown that higher tolerance against PD has indeed some underlying additive genetic components and this trait can potentially be improved through selective breeding. Norris et al. [26] estimated the heritability of 0.08 for PD resistance (0.21 on the liability scale), based on mortality data from a field outbreak due to SAV1 infection. In a controlled challenge trial, Gonen et al. [27] investigated the genetic basis of PD resistance in two different strains of Atlantic salmon (i.e., SalmoBreed and Mowi) and two different physiological stages, fry and post-smolt, against SAV3. Their estimates suggest a moderate to high heritability of 0.26–0.34 for resistance to PD. Further, using a 6 K Illumina iSelect SNP array (Centre for Integrative Genetics) as well as a low-density panel of genetic markers, Gonen et al. [27] have reported a suggestive quantitative trait locus (QTL) at the distal end of chromosome 3 (ssa03) in both populations. In addition to ssa03, their analysis also suggests putative QTL on ssa02, ssa04, ssa07, ssa14 and ssa23.

The goal of this study was to investigate the genetic architecture of PD resistance in Atlantic salmon, based on mortality data, in much greater detail than previous reports. Within a controlled infection environment and by testing approximately 4500 pedigreed fish challenged against SAV3, we first aimed to estimate the heritability of resistance against this disease. We then genotyped a subset of animals using a 55 K SNP array to identify and narrow down the genomic regions containing QTL. Finally, to identify putative candidate genes of importance within these QTL regions and also to better understand the host’s response to SAV3 infection, we compared the transcriptomic data from the animals that survived the challenge versus those that were in a moribund state early during the trial.

## Results and discussion

### Challenge and mortality

**Fig. 1:**
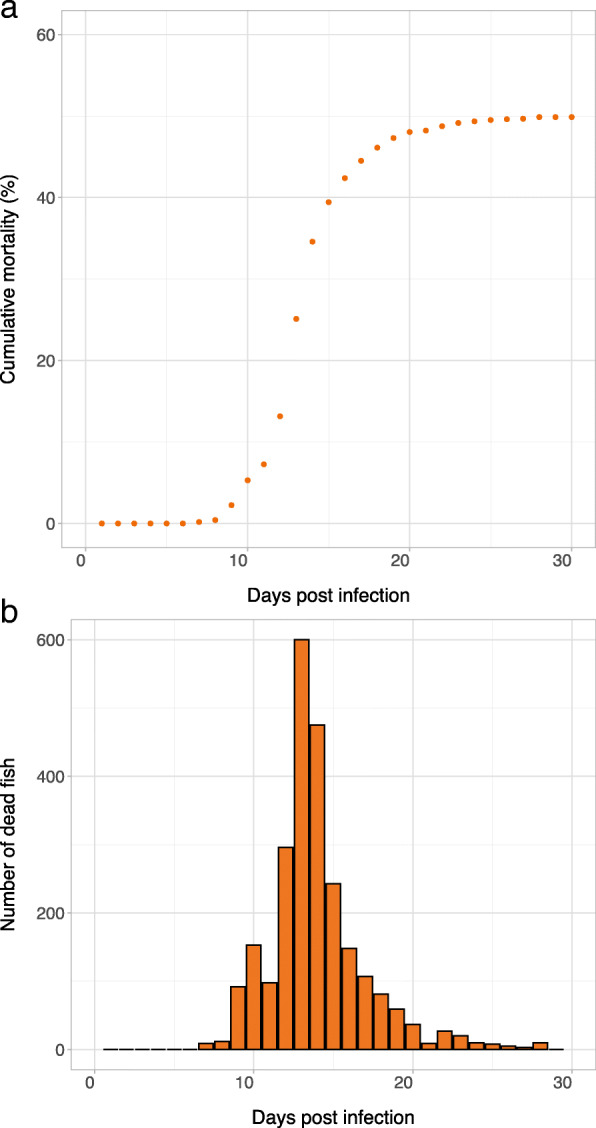
**a** The cumulative per cent mortality curve and **b** histogram of mortality profile of SAV3 infection following injection. Each bar shows the number of dead fish per day from the start (0 dpi) to the termination of the challenge (29 dpi)

A total of 4506 PIT-tagged animals with pedigree information, representing 296 full-sib families, contributed to the data following the challenge with SAV3. As expected from a SAV3 intraperitoneal injection (i.p.) model of infection [17], mortality initiated at 7 days post-infection (dpi) (Fig. 1a), and the trial was terminated after 29 dpi when the disease reached its mature phase, approximately 1 week after the mortality was plateaued. The mortality peaked at 13 dpi and started to stabilize from day 21 (Fig. 1b). At this stage, the cumulative mortality had reached approximately 47%. By the end of the experiment, a total of 2151 (approximately 48%) animals died due to PD infection.

### Heritability estimation

**Fig. 2:**
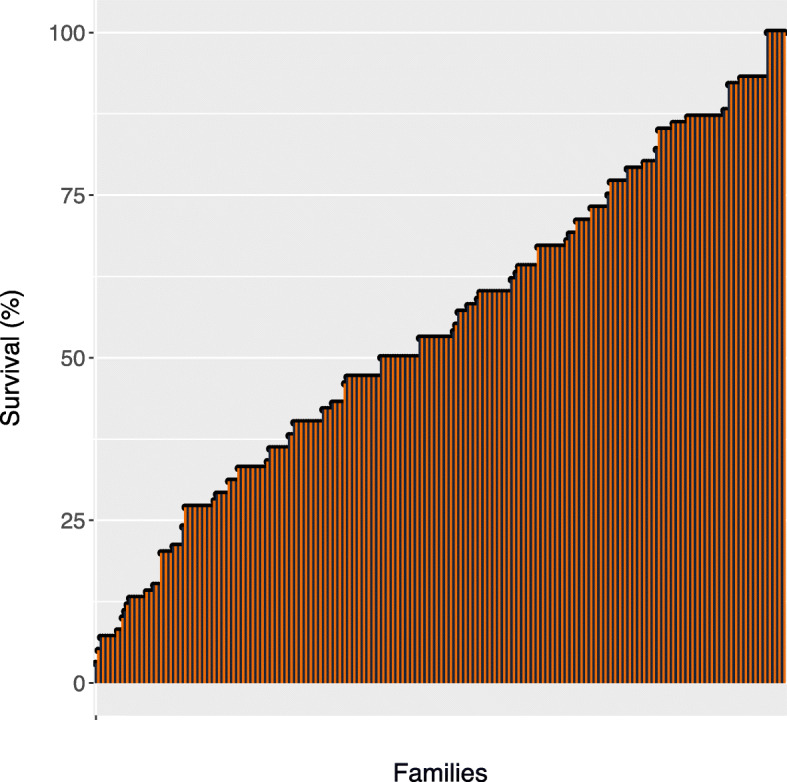
Per cent survival, per family, by the end of the challenge, at 29 dpi. A total of 296 full-sib families, shown on the x-axis and sorted based on survival rate, were used in this trial

We observed considerable variation in the rates of survival between the challenged families, ranging from 3 to 100% (Fig. 2). The pedigree-based and the SNP-based heritability were estimated to be 0.41 ± 0.017 and 0.32 ± 0.081, respectively. The heritability estimates in this study are considerably higher than the estimate reported previously, based on a field data outbreak following a SAV1 infection [26]. The estimate from the field outbreak, however, is probably an underestimation of the true heritability of the trait, considering the challenges associated with a thorough collection and early diagnosis of the infected animals, from a cage in a natural setting. Further, to accurately monitor, collect and record all mortalities that are due to a specific pathogen from the field is not a trivial task. Mortalities in the field can be due to other causes or diseases such as heart and skeletal muscle inflammation (HSMI), with manifested symptoms similar to those of PD [17]. We also have little knowledge of how various environmental factors in natural settings shape the immune response and how such a response can influence the outcome of a disease. Little is known on how sequential exposure to various biotic or abiotic elements in an environment can benefit or harm the fish and how gene by environment interactions or even the interactions between different microbes can influence the trajectory of disease progression. One might also expect substantial differences in estimates of additive genetic variance and heritability for resistance against different subtypes of the virus, i.e., SAV1 versus SAV3.

In contrast, in a controlled experiment, all mortalities, with high certainty, can be attributed to the specific pathogen of interest. Furthermore, one can monitor mortalities regularly, and various environmental parameters can be controlled and adjusted. In this regard, our experiment and estimates are more comparable and similar to those reported by Gonen et al. [27]. In their study, they challenged two different strains of Atlantic salmon, the Mowi strain, where fish were at the fry stage and the SalmoBreed strain, as post-smolts. Their estimated heritability on the binary scale ranged from 0.26–0.34. Therefore, the finding in our study supports the reported estimates by Gonen et al. [27], in that resistance to SAV3 has a moderate to high additive genetic components, and selective breeding can be an efficient and attractive tool for reducing and managing the outbreaks due to this virus.

### Genome-wide association study

**Fig. 3:**
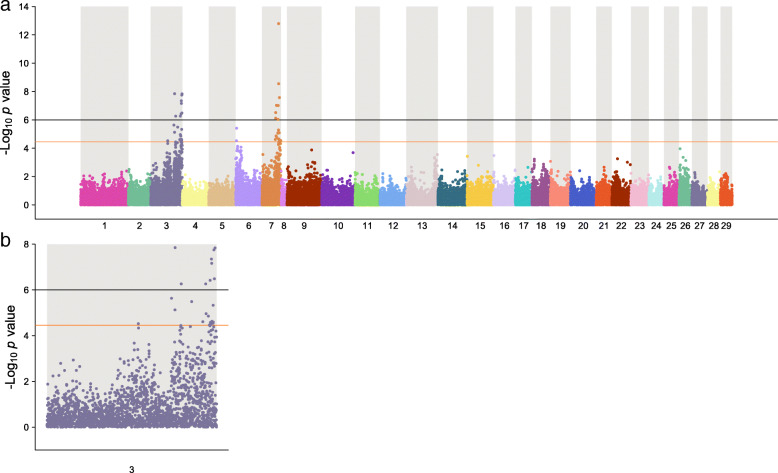
**a** Manhattan plot of association analysis to PD resistance in Atlantic salmon. The figure shows the -log_10_ (*p*-value) of the test statistics for each SNP plotted against the physical positions of the markers on the chromosomes. The black and the orange lines indicate the genome-wide and the chromosome-wide significance threshold cut-off levels, respectively. **b** Zoomed-in Manhattan plot of ssa03 showing the distribution and association of genetic markers on this chromosome

Approximately 49 K genetic markers and 903 fish, comprising of 430 dead and 473 survivors, passed all the quality control measures. The proportion of the dead and survivors among the genotyped animals was the same as that for the entire challenged population (i.e., approximately 48% mortality and 52% survivals). The genotyped fish were from 65 families, with a minimum of 10 and an average of 14 animals per family. The inflation factor was estimated to be 1.07, an indication of no confounding effect due to population stratification. A total of 17 genetic markers, nine located on chromosome ssa03 and eight on chromosome ssa07 exceeded the genome-wide significance level of *p* < 1.024e^− 06^ (Fig. 3a). The QTL on ssa03 spans a region of approximately 27 Mbp, from 63.8 to 90.8 Mbp (Fig. 3b). On the SNP chip array, there are about 655 informative genetic markers within this chromosomal segment of ssa03, which collectively explained about 31% of the additive genetic variance in our dataset (${h}_{RGH}^2$ *=* 0.100 ± 0.05). The two most significant SNPs on this genomic block are at both ends of the segment, each explaining approximately 3.4% of the phenotypic variance, suggesting the possibility of two different QTL on ssa03 (Fig. 3b; Table 1). The first SNP (ssa03: 63,829,838), is a synonymous variant, located in a member of dedicator of cytokinesis gene family (LOC106601059; Dock7). The members of this gene family are evolutionary conserved and are involved in intracellular signalling networks [28]. The second significant SNP (ssa03: 90,830,374) is an intergenic variant, flanked by a cytokine encoding gene, Metrnl (meteorin-like protein) [29]. The expression of Metrnl is regulated through products of different immune-related genes such as IL-4, IL-12 and IFN-γ, and is associated with inflammation response [29]. This gene showed an approximately 5-fold elevation in expression among animals that survived the challenge (Supplementary Table 1) compared to those that died early in the trial.

**Table 1:** Summary statistics of the QTL associated SNPs that passed the genome-wide significance threshold

Number	chr	Position	-log_**10**_***p*** value	pve	maf	DD/Dd/dd
1	3	63,829,838	7.847	3.44	0.133	682/229/8
2	3	67,000,632	6.261	2.70	0.273	467/403/49
3	3	83,391,111	6.262	2.70	0.140	667/213/19
4	3	86,046,000	6.417	2.77	0.285	466/378/72
5	3	86,785,737	7.348	3.21	0.419	303/460/154
6	3	86,938,000	7.160	3.12	0.325	426/386/105
7	3	89,417,924	7.749	3.40	0.147	665/243/14
8	3	89,963,082	6.485	2.80	0.157	648/243/22
9	3	90,830,374	7.835	3.44	0.307	448/377/94
10	7	38,641,377	6.119	2.63	0.341	363/448/80
11	7	38,651,174	6.513	2.82	0.291	448/405/65
12	7	40,642,933	7.020	3.05	0.354	356/477/87
13	7	44,154,604	6.032	2.59	0.195	597/282/38
14	7	44,449,061	7.014	3.05	0.407	355/381/184
15	7	45,525,972	12.789	5.76	0.290	466/369/81
16	7	45,556,093	8.551	3.78	0.220	565/307/49
17	7	47,761,471	7.572	3.31	0.497	221/460/215

*chr* Chromosome, *pve* Proportion of variation explained, *maf* Minor allele frequency, *DD/Dd/dd* Genotype counts

The finding of a QTL on ssa03 corroborates well with the reported QTL on the same chromosome in the two populations investigated by Gonen et al. [27]. In that study, an association on ssa03 was identified in both Mowi and the SalmoBreed strains, even though the fish were genotyped using either a sparse SNP panel (on the Mowi strain – challenged as fry) or a 4 K SNP array (SalmoBreed population – challenged as post-smolts). Further, the position of the QTL in both studies is consistent, with the most significantly associated markers located at the end of the chromosome. These independent findings, which are based on different populations, different year-classes, different challenge models and different developmental stages of the fish, validate the presence of a significant QTL on this chromosome. Our data further helps to refine and narrow down the location of the causative mutation to about 27 Mbp region of ssa03.

On chromosome ssa07, the QTL lies in a genomic region of approximately 9.12 Mbp, from 38.6 to 47.8 Mbp. There are approximately 340 informative SNPs within this segment of the chromosome, where based on the regional heritability estimates, they accounted for approximately 33% of the additive genetic variation (${h}_{RGH}^2$ *=* 0.106 ± 0.05). The most strongly associated SNP on this genomic block is an intergenic SNP (ssa07: 45,525,972), explaining about 5.8% of the variation (Table 1). This SNP is in a short distance from the 3′ untranslated region of an uncharacterized gene with a putative protein structure similar to the products of extensins. In plants, extensin proteins, through the strengthening of the cell walls, play an essential role in the defence mechanism against pathogens, preventing tissue damages and are involved in wound responses [30–32]. In our transcriptome data, the products of this gene showed a 1.75-fold increase in expression among the survived animals (data not shown). On the downstream of this SNP, there are also a few genes of interest, including fibroblast growth factor receptor oncogene partner 2 (FGFR1OP2), a gene with potential roles in the wound-healing pathway [33, 34]. By the end of the experiment, the transcription level of this gene increased by 2-fold (data not shown). Gonen et al. [27] reported a suggestive QTL on ssa07, detected only on the Mowi strain. However, due to the low resolution of their mapping SNP panel, it is not possible to localize the position of QTL and make a comparative assessment with the QTL on ssa07 identified in this work. Further, Gonen et al. [27] did not find any association on this chromosome in the SalmoBreed strain, possibly due to QTL not segregating in the 2009 year-class of this population.

While no marker on ssa06 passed the genome-wide significant threshold, two markers on the p arm of this chromosome (ssa06: 3,319,000 and ssa06: 3,846,000) passed the chromosome-wide significance level. The proportion of phenotypic variation explained by each of these markers is about 2%. This finding is interesting, as the p arm of ssa06 shares homeology (i.e., shared ancestry due to the whole genome duplication event at the base of all extant salmonids) with the q arm of ssa03 [35], where we have mapped the QTL on chromosome 3. Therefore, one might speculate the possibility of a duplicated QTL on the homeologous chromosomal regions of these chromosomes. At the same time, we cannot dismiss the possibility of this peak being a by-product of miss-assembly in the reference genome, where some duplicated contigs or scaffolds from ssa03 have mistakenly collapsed with sequences on ssa06. This idea is further supported by relatively lower average LD (*r*^*2*^) within the QTL segment of ssa06 (0.46) compared to that of ssa03 (0.54).

### Analysis of transcriptomic data

**Fig. 4:**
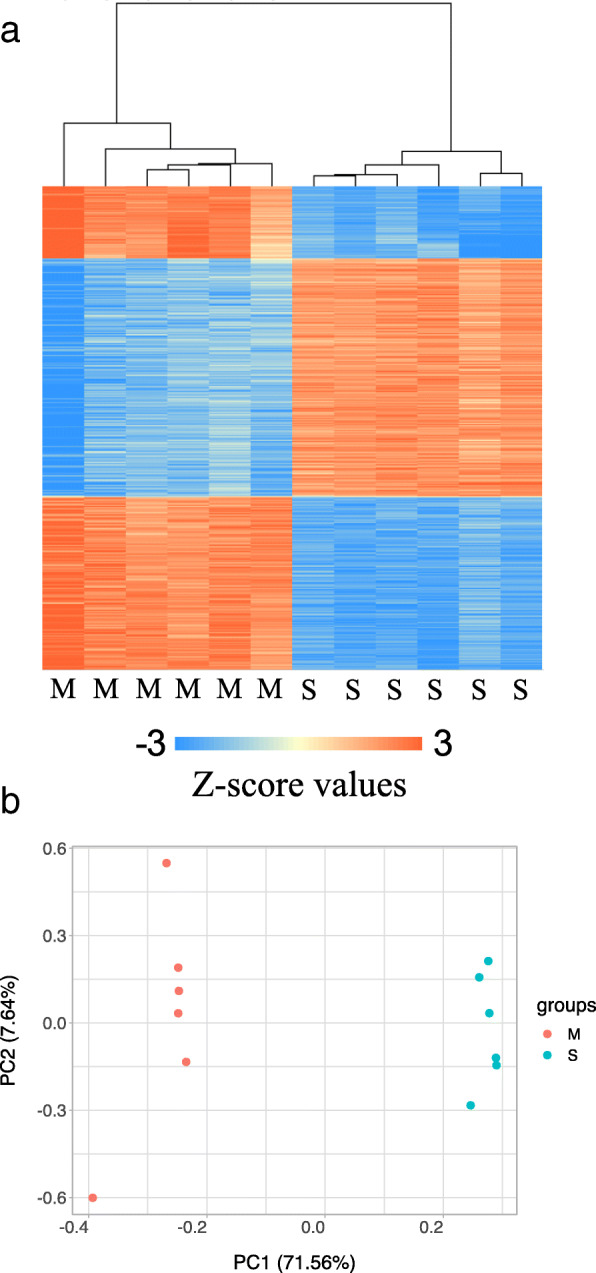
**a** Heatmap of the transcriptome sequence data between the moribund animals (M) at 11 dpi and the animals that survived (S) the challenge. The x-axis represents animals that were sampled during the two stages. The y-axis shows the expression profiles of differentially expressed genes between the two groups (Supplementary Table 1). **b** Principle component clustering of the same transcriptome data

We obtained, on average, 15 million paired-end reads transcriptome sequence data, per animal, from the apex of the heart. Following trimming and filtering the low-quality reads, sequences with multiple hits against the genome and removal of rRNA and Illumina adapter contaminations, approximately 10 million uniquely aligned paired-end reads per fish retained for assessment of the gene expression profiles. Both cluster and principal component analysis (PCA) of the gene expression data, separated and grouped animals based on the survival status of the fish (Fig. 4a and b), confirming significant changes in the transcriptional regulation of a vast number of genes during infection and between the moribund and survived animals. In total, we identified 7431 differentially expressed transcripts between the two groups. Approximately, half of these transcripts showed a higher level of expression in the animals that survived the challenge (3480 transcripts) while the other half (3951 transcripts) had higher expression in the moribund animals (Supplementary Table 1). The functional enrichment analysis of these gene sets suggests a broad range of genes with different functional properties to be enriched within each group (Supplementary Table 2).

The transcriptome of the most severely sick fish had signatures of high levels of expression of genes associated with innate immune response and IFN-related antiviral genes. On the other hand, fish, which survived till the termination of the challenge, exhibited higher expression of genes specific for adaptive immunity (Supplementary Table 1). Similar differences exist in Atlantic salmon with the early and late mortality following infection with the infectious salmon anaemia virus – ISAV [36]. As previously reported, the expression of genes involved in the innate antiviral responses shows a strong correlation with loads of SAV [17], and therefore, the observed transcriptional differences between the moribund and the survived fish, in this study, were expected.

The PRR machinery is essential for triggering various components of the immune response. In an Atlantic salmon, infected with a virus, RNA helicase Rig-I is usually among the genes with the most active response. In our study, this gene had significantly higher expression in moribund fish. Across the animals that survived the infection, we found significantly higher levels of expression in a Toll-like receptor (TLR13) and the NOD-like receptor gene (NLR family member X1), both of which being part of the PRR. In response to a viral infection, TLR13 can activate a MyD88-dependent pathway, an essential element for signalling within the immune cells, resulting in the activation of nuclear factor κB (NF-κB) and type I IFN via interferon regulatory factor 7 (IRF7) [37]. IRF4 and IRF8, which found to have higher expression in the resistant animals, control termination of pre-B-cell receptor signalling, and therefore, promote differentiation to small pre-B-cells undergoing light-chain gene rearrangements [38]. Besides, a panel of genes involved in the regulation of IFN-γ, a member of the type II class of IFN with a critical role in T-cell mediated adaptive immunity, had elevated their expressions in the animals that survived the challenge (AXL, CD2 (LOC106575562), CD226, CD276 (LOC106587232), CD3E, HMGB1, IRF8 (LOC106562284), TNFR5 (LOC106575195), ZFPM1; Supplementary Table 1).

Chemokines are important in triggering inflammation in virus-infected tissues. Many genes involved in the chemokine-mediated signalling showed significantly higher levels of expression in the moribund animals. Some examples are chemokine ligand 17 (CCL17; LOC106613702), chemokine ligand 19 (CCL19; LOC106563358), chemokine receptor-like 2 (CCRL2; LOC106600445), chemokine ligand 1 (CXCL1; LOC106580387), chemokine ligand 11 (CXCL11; LOC106564720), chemokine ligand 4 (CCL4; LOC106600447), chemokine ligand 25 (CCL25; LOC106613704) and chemokine ligand 20 (CCL20; LOC106570861). These genes play essential roles in the recruitment of leukocytes to sites of infection and inflammation and guiding the lymphocytes throughout the body [39]. It is important to note that excessive recruitment of chemokine gene products can result in damages to the tissue. For a number of these genes, we identified two or more gene copies, located on different chromosomes, with significant expression profile differences between the moribund and the survived animals. For example, three copies of CCL19, on ssa01, ssa11 and ssa14, all had higher levels of expression at 11 dpi. At least two of these chromosomes, ssa01 and ssa11, are known to share ancestral homeologous regions [35]. Similarly, duplicated copies of CCL25 on the homeologous segments of ssa10 and ssa16 and CXCL11 on the homeologous regions of ssa02 and ssa12 exhibited elevated levels of expression in animals with early mortality.

The most significantly down-regulated gene, not only among the immune-related genes but within the entire dataset, is the complement component C1q-like protein 2, with almost no expression among the animals that survived the challenge (log_2_-fold change of 9.877). The role of this gene in salmonids, which have also shown to be down-regulated in several other viral infections, is not very well understood. However, it is unlikely that its lack of expression is a sign of suppression of a classical complement pathway, but rather an indication of a connecting bridge between the innate and the acquired immunity [40, 41]. Such a hypothesis is further supported considering that several other complement components, such as C1S (LOC106605669), C1QC, CLU (LOC106581046), C6 and CD55, had increased their levels of expression by the end of the challenge.

In contrast, the gene with the highest fold-change increase, by the end of the experiment, was a gene with an eotaxin-like product. Eotaxins have an essential function in stimulating and chemotaxis of eosinophil, a specialized pro-inflammatory white blood cell, [42, 43], but are also crucial in the recruitment of helper T-cells [44], mast cells [45], and directing the killer lymphocytes and monocytes to the sites of inflammation [46]. We further found strong evidence for the activation of specific adaptive immunity genes among survivors. As a few examples, several genes associated with cellular immune response and T-cell activation (CD2 (LOC106575562), CD3E and CD28 (LOC106575093) have significantly elevated expression in the fish that survived the challenge. Similarly, CD74 (LOC106604844), which plays a crucial role in MHC class II antigen processing through stabilizing peptide-free class II alpha/beta heterodimer, was also found to have been expressed about 2-fold higher in these animals. Tumour necrosis factor ligand superfamily member 11 (TNFSF11), a gene involved in stimulating naïve T-cell proliferation that regulates the interactions between T-cell and dendritic cells, was also expressed 2.5-fold higher in survived animals. Since the peak of acquired immune responses in SAV infected salmon usually occurs after three to 4 weeks post-infection, the transcriptional differences between the moribund and survivors were also likely affected by the sampling time. However, the ability to survive until the development of adaptive immunity against SAV may be a critical factor that determines resistance against PD.

### Candidate immune-related genes in the QTL regions

The QTL region on ssa03 contains more than 900 annotated genes of which we found 135 with different expression patterns between the two groups. On ssa07, the genomic segment containing QTL has about 156 expressed genes, of which 62 had significantly modified their expression profiles. A heatmap of genes with putative immune-related functions and different expression profiles within these QTL segments are presented in Supplementary Figure 1a and b. These genes are candidates that can potentially affect an animal’s resistance to PD infection. The most notable of these genes, however, are multiple transcripts from the immunoglobulin heavy chain (IGH) locus on ssa03 and the immunoglobulin light chain locus on ssa07. The transcripts exhibited 4 to 20 times higher levels of expression in the animals that survived the test.

The immunoglobulin molecules in Atlantic salmon consist of an assemblage of two heavy and two light chains, made up of constant and variable domains. The duplicated IGH loci in Atlantic salmon reside on the homeolog of two chromosomes, ssa03 (IGH-B) and ssa06 (IGH-A) [47]. The initial size estimation of IGH-B locus suggested a genomic region of 700 Kb [47], but in the current assembly of the Atlantic salmon genome, the clusters of IGH segments span a much larger region, covering from 77.5 to 79.3 Mb. In addition to the constant segments (CH) of all three types of IG (i.e., D, M and T), the IGH-B locus contains 28 DH (diversity) genes, eight JH (junction) genes and numerous VH (variable) genes, of which 36 genes are functional. In our data, about 92% of the IGH transcripts exclusively map to IGH-B, and almost all transcripts encode an immunoglobulin type M (IgM). On ssa07, a significant increase in the expression of two immunoglobulin constant (IGKC) and three variable domains (IGKV) were detected (Supplementary Figure 1b). In B-cells, antibodies are expressed and assembled through somatic recombination of variable segments. Recombination is followed by enzymatic insertion and deletion of nucleotides in the variable region, generating a broad diversity of antibodies, which can recognise a diverse array of antigens. The findings presented in the current study, and the fact that the two QTL are in close genomic proximities to the immunoglobulin loci, suggest them as likely candidates for conferring higher resistance against PD virus in this species.

Although there are various lines of evidence regarding the crucial role of antibodies in defence against viruses, the early activation of antiviral immunity and inflammatory responses does not necessarily prevent the propagation of the pathogen and the subsequent development of the disease. Clearance of pathogens and disease symptoms usually take place shortly after the initiation of the adaptive immunity [17, 36]. In the case of PD, B-cells can combat SAV in different ways. Marked increase of the IG transcripts in the infected hearts is an indication of the recruitment of B-cells, which can either produce antibodies locally or act as phagocytic cells [48]. The membrane-bound form of salmon IgM lacks the third exon of the constant region. Considering that the expression levels of transcripts from all the exons of the constant regions were similar in our dataset, one might expect that the secreted form of IgM was predominant. Immunization of Atlantic salmon with a vaccine can cause a marked increase of polyspecific antibodies, which precede the appearance of antigen-specific antibodies [49]. Mutations in IGH locus may affect the time-course of antibody responses, their antigen-binding properties or the size and diversity of antibodies repertoire. Each of these factors can be important for conferring higher resistance against SAV. As it stands, however, our knowledge of the genetic variations in the immunoglobulin loci is limited, and the coverage of genetic markers spanning these regions in genomic studies conducted so far in Atlantic salmon has not been optimal. Further, considering the duplicated nature of the salmon genome and the repetitive structure of these loci, there is a need for additional sequencing and genome assemblage, fine mapping, and a basic understanding and comparative analysis of these genes across different populations.

## Conclusions

The findings reported in this study provide knowledge, necessary for helping us better understand the genetic basis of resistance against PD. First, our data confirm that PD resistance has a high heritable, additive genetic component. The estimated heritability of 0.41 on the observed binary scale, is among the highest estimates for any disease resistance in Atlantic salmon. One might also expect this finding to be irrespective of the mode of infection, as recently we have shown that resistance against SAV3 is not influenced by the means at which the virus enters the host, whether it is through injection or via cohabitation [50]. Second, it seems that the majority of the genetic variation for PD survival can be explained by two significant QTL, one located on ssa03 and the other on ssa07. These QTL collectively explained more than 60% of the additive genetic variation, in our population, respectively. Therefore, it would be possible to both implement and complement a breeding program for improving PD resistance using marker-assisted selection based on information within these QTL regions. Third, we have narrowed down the location of QTL on each of these chromosomes and complemented genomic analysis with transcriptomic data. Within the QTL containing segments, we have identified several candidate genes with potential impacts on an animal’s response to PD infection. In particular, a locus with multiple components of immunoglobulin heavy chain genes on ssa03 and a few immunoglobulin chain loci on ssa07 are of specific interest. Notably, resistance to PD seemed to be associated with increased transcription of antibodies producing secretory IgM. The finding of the potential role of antibodies in both genetic and acquired resistance against PD provides an attractive platform for close collaboration and synergy in vaccine development and breeding programs.

## Methods

### SalmoBreed population and challenge test

In November 2015, 4506 PIT-tagged (passive integrated transponder) smolts were transported from NOFIMA MARINE AS in Sunndalsøra, Norway (https://nofima.no/en/) to the challenge facility in VESO Vikan (https://www.veso.no/about-us; Namsos, Norway). The group consisted of all families from the entire 2015 year-class of the SalmoBreed nucleus, approximately 15 individuals per family, to assure reliable estimates of genetic parameters [51]. At the parr stage, all fish were PIT-tagged, weighted and reared in the same tank till smoltification when they were transferred to the challenge facility. Following arrival at the VESO Vikan, fish were kept in a single tank and acclimatized at 12 °C brackish water and 24:00 h light regime for 8 days. After acclimatization, using a common-garden approach, all fish were i.p. injected with 0.1 mL of a cell culture containing SAV3 at a concentration of 10^4.8^ TCID_50_ per mL and the challenge continued in the same tank. The SAV3 isolate, R-1-2007 (GenBank: LT630447.1), used in this study, is one of the most pathogenic forms of the virus found in Norway [52]. Random mortalities were collected throughout the trial and examined for assessing the cause of death. The experiment aimed to target 50% mortality, in order to maximize the phenotypic variation expected from a case-control study (i.e., var. (trait) = *p* × (1 - *p*), where *p* is the frequency of surviving animals). At the end of the experiment, approximately 4 weeks after the infection when the mortalities stabilized, the remaining fish were euthanized with an overdose of anaesthesia (100 mg/L, Finquel®Vet, ScanAqua AS, Årnes, Norway).

### Pedigree-based genetic estimations

The pedigree-based variance components, i.e., the additive genetic (${\sigma}_a^2$), common-environmental family (${\sigma}_c^2$) and residual variances (${\sigma}_e^2$), were estimated using a threshold model implemented in the THRGIBBS1F90 module from the BLUPF90 package [53]. The Gibbs sampling scheme was run for 120,000 iterations with the first 20,000 discarded as burn-in and then set to save every 100th sample. The model used was as follows:

$$ \boldsymbol{y}=\mathbf{X}\boldsymbol{b}+{\mathbf{Z}}_{\mathbf{1}}\boldsymbol{c}+{\mathbf{Z}}_{\mathbf{2}}\boldsymbol{u}+\boldsymbol{e} $$

where ***y*** is a vector of PD survival status, recorded as a binary trait, **X**, **Z**_**1**_ and **Z**_**2**_ are design matrices assigning the phenotype to the fixed effect, tag weight (***b***), and the random variables, the common-environmental full-sib family prior to tagging (***c***) and the animal (***u***) effects. The residuals are defined in the ***e*** vector. The random effects are assumed to have the following distributions, $\mathrm{c}\sim N\left(0,\mathbf{I}{\sigma}_c^2\right)$, $u\sim N\left(0,\mathbf{A}{\sigma}_u^2\right)$ and $e\sim N\left(0,\mathbf{I}{\sigma}_e^2\right)$*,* where **I** is an identity matrix, **A** is the additive genetic numerator relationship matrix, ${\sigma}_{\mathrm{c}}^2$, ${\sigma}_{\mathrm{u}}^2$ and ${\sigma}_e^2$ are the variances of full-sib family effect, additive genetic and residual respectively. The pedigree-based heritability was estimated as:

$$ {h}^2=\frac{\sigma_a^2}{\sigma_a^2+{\sigma}_c^2+{\sigma}_e^2} $$

### Genotyping and genotype quality assessment

Adipose fin-clip from 930 of the challenged animals, representing 65 families, were collected throughout the trial. With an average of 15 fish per family, the choice of family selection was set to maximize the genetic diversity, based on the pedigree information, while utilizing the within-family genetic variations [51, 54–56]. DNA extraction and genotyping were performed by IdentiGEN (https://identigen.com/; Dublin, Ireland). We used a 55 K custom made Affymetrix Axiom array and the Affymetrix Axiom analysis suite software to genotype the fish and call the SNPs. Additional genotype quality measures and filtering were applied using the SNP & Variation Suite v8.8.3 (SVS; Golden Helix Inc., Bozeman, MT, USA www.goldenhelix.com). Samples and SNPs with call rates < 98%, Hardy-Weinberg *p*-value (Fisher’s exact test) < 10–10 or minor allele frequency < 0.05% were excluded, leaving 903 animals and about 49 K markers for the subsequent genomic analysis.

### Estimation of SNP-based heritability and the QTL effect size

The restricted maximum likelihood method, implemented in the GCTA software [57], was used to estimate variance components and calculate the SNP-based heritability. We applied a model with variables similar to the one used for estimating the pedigree-based genetic parameters except that *i.* the gender effect, predicted according to the Y-specific linked markers [58], was also included into the model and *ii.* the **A** matrix was replaced with the genomic relationship matrix (i.e., the **G** matrix), computed as described previously [57, 59]. We then used the software package DISSECT to estimate the proportion of genetic variation that is explained by the genomic loci harbouring the QTL (${h}_{RGH}^2$*)* [40, 41] by first dividing SNPs between the genetic markers located within the regions of interest (**R**_*k*_) and those covering the rest of the genome (**G**_*k*_). The genomic relationship matrices (${\mathbf{G}}^{R_k}$ and ${\mathbf{G}}^{G_k}$) and variances (${\sigma}_{g^{R_k}}^2\ \mathrm{and}\ {\sigma}_{g^{G_k}}^2$) were then computed and fitted using the genetic markers within each group [41].

### Genome-wide association study

SVS was used to conduct the genome-wide association study (GWAS) using a mixed linear model. The methodological details of the association analysis have been described previously [59]. In brief, we first pruned the quality-controlled markers (window size 40, window increment 5 and *r2* threshold 0.5) to construct an n × n genetic distance relationship matrix based on the identity-by-state (IBS). The relationship information was then used to perform a single-locus mixed-model association analysis, using the full set of quality-controlled markers, by applying Efficient Mixed-Model Association eXpedited (EMMAX) [60, 61]. The model used for the analysis can generally be described as:

$$ \boldsymbol{y}=\mathbf{X}\boldsymbol{b}+{\mathbf{Z}}_{\mathbf{1}}\boldsymbol{c}+{\mathbf{Z}}_{\mathbf{2}}\boldsymbol{u}+{\mathbf{Z}}_{\mathbf{3}}\boldsymbol{a}+\boldsymbol{e} $$

where ***y*** is the vector of survival scores, **X**, **Z**_**1**_, **Z**_**2**_ and **Z**_**3**_ are the incidence matrices assigning phenotype to the vectors of fixed effects (***b***), i.e., the animal weight at tagging and gender, the common-environmental family effect (***c***), additive genetic effect (***u***; $\mathrm{Var}(u)={\sigma}_u^2\mathbf{K};\mathbf{K}=\mathrm{IBS}\Big),$ allele substitution effect of the SNP (***a***), assigned to the matrix for marker genotypes and the residual (***e***;$\mathrm{Var}(e)={\sigma}_e^2\mathbf{I};\mathbf{I}=$ identity matrix). The genome-wide and chromosome-wide significance thresholds were set as 0.05/*NG* and 0.05/*NC*, respectively, where *NG* is the total number of quality-controlled genetic markers in the study, and *NC* is the number of SNPs per [62, 63]. Although EMMAX only performs standard linear regression, even on binary data, its suitability for assessing case-control studies have been thoroughly investigated and validated [60]. Nonetheless, we further compared its results with analysis based on a logistic mixed model, using the GMMAT R package [64], where we applied a logit link function for binary traits and found the results to be almost identical.

We estimated the linkage disequilibrium (LD) within the QTL regions, for SNP pairs separated by up-to 100 kb, using the *r*^*2*^ function in plink v1.9 [65]. The inflation factor (lambda) was computed as the ratio of the median of the observed Chi-squared test statistics to the expected value, in the absence of stratification (0.456) [66]. The proportion of the phenotypic variation explained by each SNP was calculated as:

$$ pve=\frac{mrss_{h0}-{mrss}_j}{mrss_{h0}} $$

where *mrss*_*h0*_ is the Mahalanobis total sum of squares, proportional to the variance of the data, and *mrss*_*j*_ is the Mahalanobis residual sum of squares for marker *j* [61, 67, 68].

### Analysis of the transcriptome sequence data

We investigated the host transcriptomic response to SAV3 infection by collecting the apex of the heart from six challenged fish at a moribund state during the early stage of the challenge (11 dpi) and six individuals that survived till the termination of the experiment (29 dpi). Heart rather than pancreas was chosen as the target tissue, as it is usually more damaged due to infection and it is less affected by cross-tissue contamination during sampling [12, 50]. The collected tissue samples were immediately immersed in RNALater (Ambion) and stored in − 20 °C. The Qiagen RNeasy Plus mini kit was used for extracting total RNA. Library preparation of and sequencing were performed by the Norwegian High-Throughput Sequencing Centre in Oslo, using the standard protocols outlined by Illumina (www.illumina.com). Sequencing was done on an Illumina HiSeq 4000 platform as paired-end (PE) 150 bp reads.

The initial sequence quality assessment was done by fastQC (www.bioinformatics.babraham.ac.uk/projects/fastqc/). The sequencing adapters and low-quality nucleotides were removed by Trimmomatic (v.0.36) [69]. The remaining sequences were then aligned to the salmon reference genome assembly ICSASG_v2 (https://www.ncbi.nlm.nih.gov/assembly/GCF_000233375.1/) using TopHat (v.2.0.13) [70]. Only reads with a single hit against the genome were used for gene expression assessment. The transcriptome profile information was assessed against the RefSeq annotation, GCF_000233375.1 (https://www.ncbi.nlm.nih.gov/assembly/GCF_000233375.1). The expression data were scaled using the median of the geometric means of fragment counts across all samples [71]. Estimation of the magnitude of gene expression and evaluation of differential levels of transcription was performed by Cuffdiff. For a gene to be assigned as differentially expressed, we set a minimum of 1.5-fold change in expression and an adjusted *p*-value of less than 0.05. More information regarding details of sequence handling, assessment and analysis have been outlined previously [72, 73].

## Supplementary information

**Additional file 1: Supplementary Figure 1.** Heatmap of the immune-related genes, with different expression profiles between the moribund (M) and survived animals (S) within the QTL containing regions of **a**. ssa03 and **b**. ssa07.

**Additional file 2: Supplementary Table 1.** Differentially expressed genes between the moribund animals at 11 dpi (sample_1) and animals that survived the challenge (sample_2). **Supplementary Table 2.** Functional enrichment analysis of differentially expressed genes between the moribund animals at 11 dpi and animals that survived the challenge.

## Data Availability

All the sequence data have been submitted to the short sequence reads archive under the SRA accession number PRJNA590166. The GWAS summary statistics data for all the key findings of the study is available in Table 1. The raw genotype data is not publicly available as it is the property of a commercial enterprise. Requests to access the raw genotype material should be directed to the corresponding author.

## Abbreviations

dpi: Days post-infection
GWAS: Genome-wide association study
FPKM: Fragments per kilobase of transcript per million mapped reads
HSMI: Heart and skeletal muscle inflammation
IBS: Identity-by-state
i.p.: Intraperitoneal injection
ISAV: Infectious salmon anaemia virus
LD: Linkage disequilibrium
Mbp: Mega base-pair
PCA: Principal component analysis
PD: Pancreas disease
PRR: Pattern recognition receptor
QTL: Quantitative trait locus
SAV: *Salmonid alphavirus*
SNP: Single nucleotide polymorphism
