# Supplementary figures and images for: Identification of genetic loci associated with higher resistance to pancreas disease (PD) in Atlantic salmon (Salmo salar L.)
Source: BMC Genomics. 2020 Jun 3;21:388. doi: 10.1186/s12864-020-06788-4 (PMC7268189; doi:10.1186/s12864-020-06788-4)

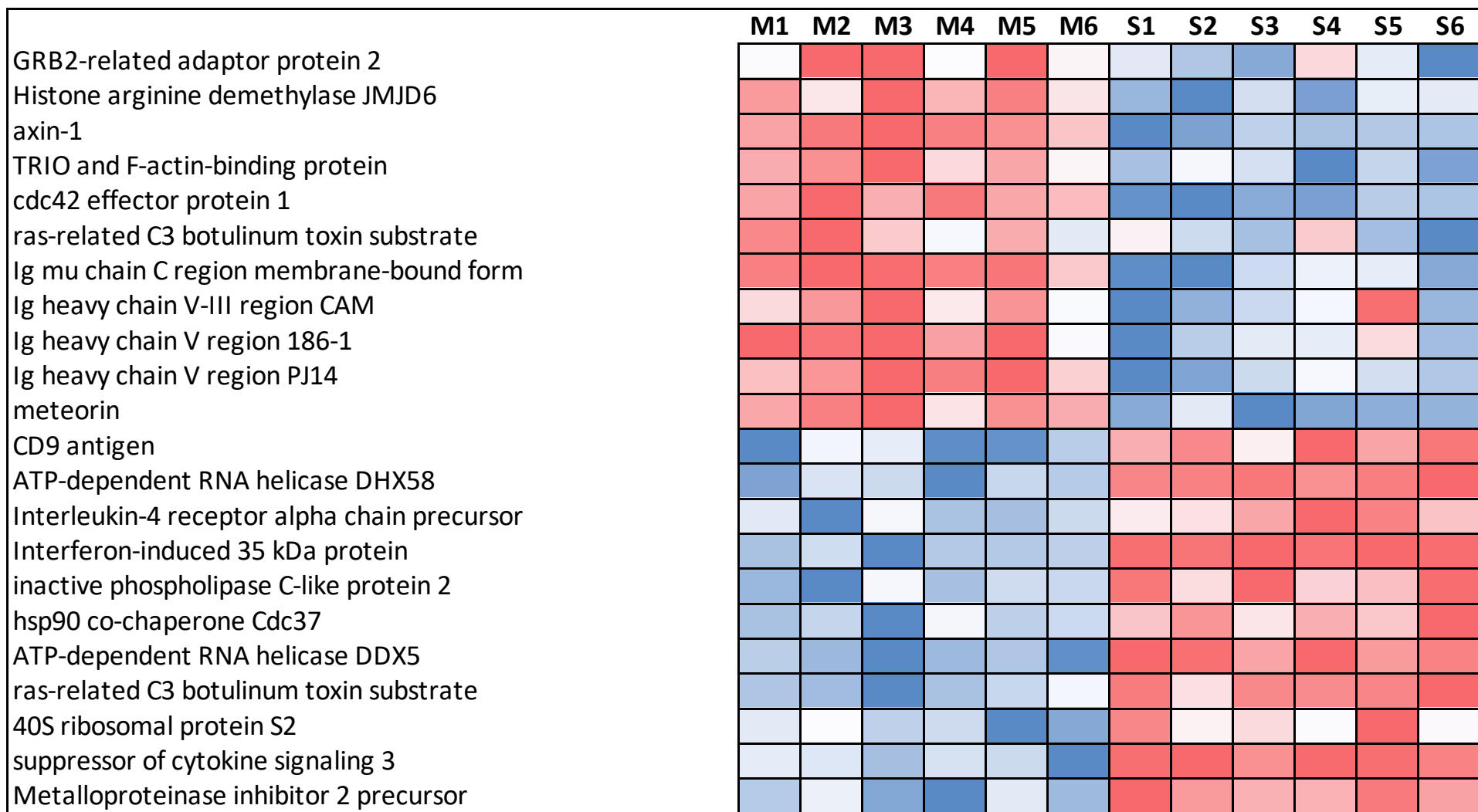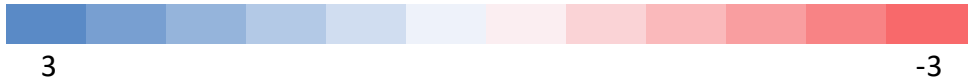

Supplementary Figure 1a

Supplement: Supplementary file 1 — Additional file 1: Supplementary Figure 1. Heatmap of the immune-related genes, with different expression profiles between the moribund (M) and survived animals (S) within the QTL containing regions of a. ssa03 and b. ssa07. [file 12864_2020_6788_MOESM1_ESM.zip › Supp.Figure1a.pdf]

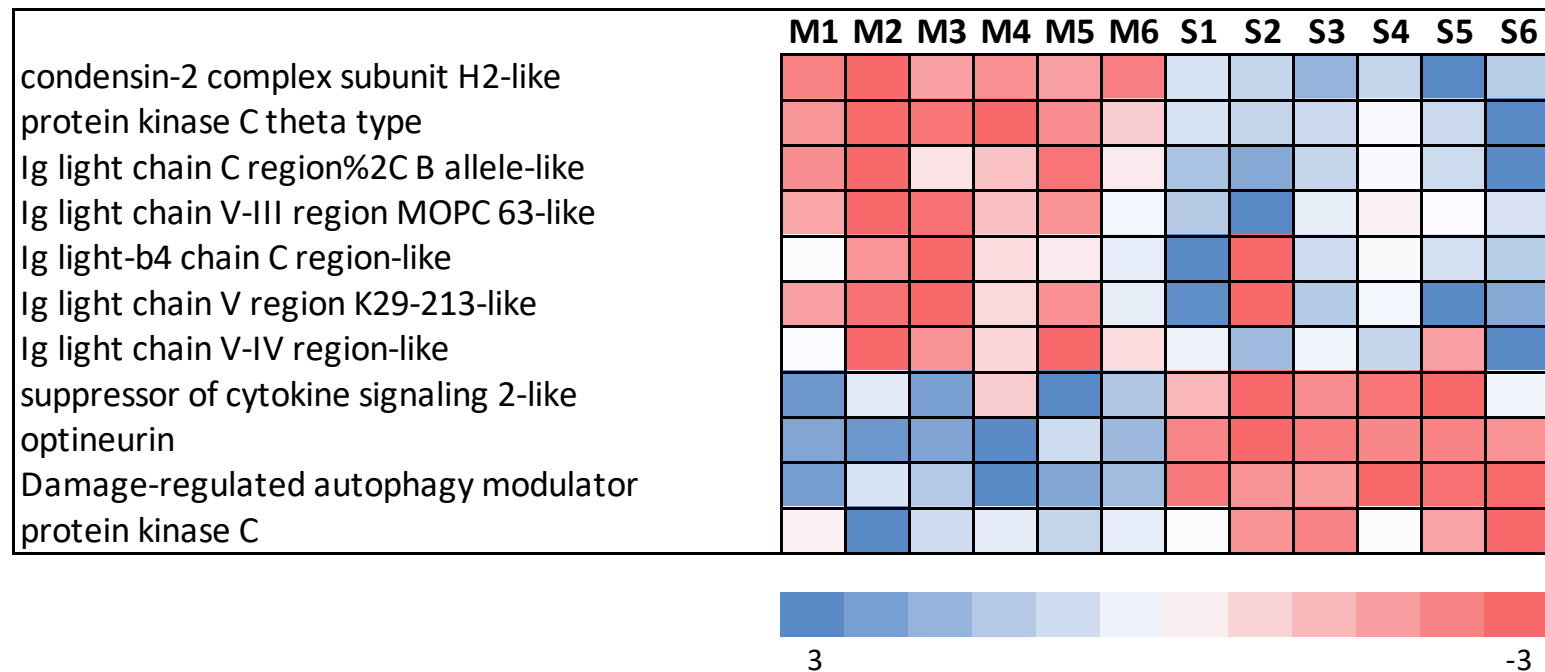

Supplementary Figure 1b

Supplement: Supplementary file 1 — Additional file 1: Supplementary Figure 1. Heatmap of the immune-related genes, with different expression profiles between the moribund (M) and survived animals (S) within the QTL containing regions of a. ssa03 and b. ssa07. [file 12864_2020_6788_MOESM1_ESM.zip › Supp.Figure1b.pdf]
